# Supplementary material for: Identification of photoperiod-induced specific miRNAs in the adrenal glands of Sunite sheep (Ovis aries)
Source: Front Vet Sci. 2022 Jul 22;9:888207. doi: 10.3389/fvets.2022.888207 (PMC9354845; doi:10.3389/fvets.2022.888207)
Supplement: Supplementary file 1 [file Data_Sheet_1.ZIP › Supplementary file/miRDeep2 file.zip!/miRDeep2 file/Know_Structure/LP42A3/oar-mir-23a.pdf]

miRBase precursor : oar-mir-23a
Total read count : 50517
oar-miR-23a read count : 25298
oar-miR-23b read count : 25217
remaining reads : 2

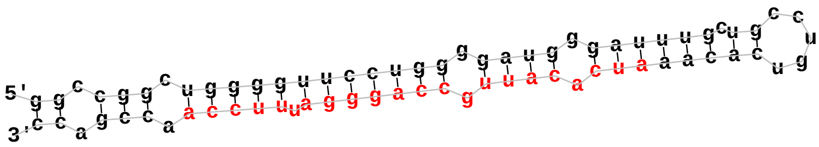

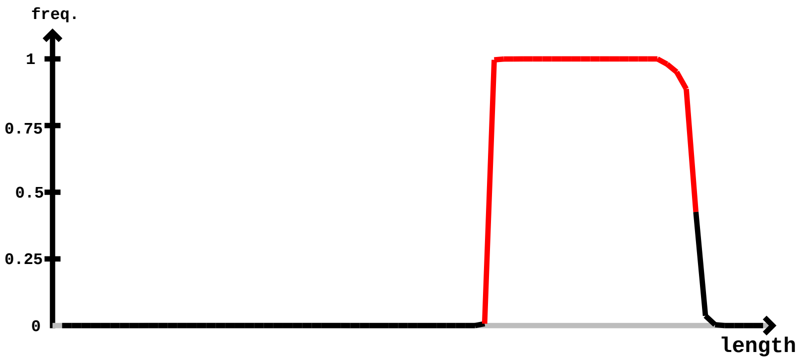


```
                                            oar-miR-23a
                                            oar-miR-23b
5'- ggccggcugggguuccuggggaugggauuugcugccugucacaaaucacauugccagggauuuccaaccgacc   -3'    exp
    ((.(((.((((((((((((.((((.((((((.((.....)))))))).)))).))))))).))))).))).))   reads   mm   sample
    ........gggguuccuggggaugggauu............................................   1       0    seq
    ........gggguuccuggggaugggauuu...........................................   1       0    seq
    ..........................................aaaucacauugccagggauu...........   1       0    seq
    ...........................................aaucacauugccagggauu...........   4       0    seq
    ...........................................aaucacauugccagggauuu..........   7       0    seq
    ...........................................aaucacauugccagggauuuc.........   12      0    seq
    ...........................................aaucacauugccagggauuucc........   99      0    seq
    ...........................................aaucacauugccagggauuucca.......   48      0    seq
    ...........................................aaucacauugccagggauuuccaa......   3       0    seq
    ...........................................aaucacauugccagggauuuccaac.....   1       0    seq
    ............................................aucacauugccagggau............   3       0    seq
    ............................................aucacauugcUagggauu...........   2       1    seq
    ............................................aucacauugccagggauu...........   483     0    seq
    ............................................aucacauugccagggauuu..........   733     0    seq
    ............................................aucacauugccagggauuuc.........   1595    0    seq
    ............................................aucacauugccagggauuucc........   11532   0    seq
    ............................................aucacauugccagggauuucca.......   9778    0    seq
    ............................................aucacauugccagggauuuccaa......   836     0    seq
    ............................................aucacauugccagggauuuccaac.....   71      0    seq
    .............................................ucacauuUccagggauu...........   1       1    seq
    .............................................ucacauugccagggauu...........   6       0    seq
    .............................................ucacauugccagggauuu..........   2       0    seq
    .............................................ucacauugccagggauuuc.........   3       0    seq
    .............................................ucacCuugccagggauuuc.........   1       1    seq
    .............................................ucacauugccagggauuucc........   29      0    seq
    .............................................ucacauugccagggauuucca.......   22      0    seq
    .............................................ucacauugccagggauuuccaa......   6       0    seq
    .............................................ucacauugccagggauuuccaac.....   9       0    seq
    ..............................................cacauugccagggauuucc........   1       0    seq
    ..............................................cacauugccagggauuucca.......   1       0    seq
    ...............................................acauugccagggauuucc........   6       0    seq
    ...............................................acauugccagggauuucca.......   3       0    seq
```
